# Advancing Medical Diagnostics: Rapid, Label-Free Detection and Differentiation of Shiga Toxin Variants in Human Serum Using a Cost-Effective PCA-Assisted SERS Platform

**DOI:** 10.1021/acsami.5c18171

**Published:** 2025-11-07

**Authors:** Alessia Milano, Amalia D’Avino, Valentina Marchesano, Domenico Sagnelli, Massimo Rippa, Bryan Guilcapi, Lu Zhou, Elisa Varrone, Giorgia Rossi, Maurizio Brigotti, Gianluigi Ardissino, Stefano Morabito, Lucia Petti

**Affiliations:** 1 Institute of Applied Sciences and Intelligent Systems “E. Caianiello” CNR, Pozzuoli 80078, Italy; 2 Department of Engineering, University of Naples “Parthenope”, Centro Direzionale Isola C4, Napoli 80143, Italy; 3 Shandong First Medical University & Shandong Academy of Medical Sciences, Jinan 271016, China; 4 Dipartimento di Scienze Mediche e Chirurgiche, Sede di Patologia Generale, Università di Bologna, Bologna 40126, Italy; 5 Fondazione IRCCS Ca’ Granda Ospedale Maggiore Policlinico, Milano 20122, Italy; 6 Department of Food Safety, Nutrition and Veterinary Public Health, Istituto Superiore di Sanità, Rome 00161, Italy

**Keywords:** nanoparticles, Shiga toxin, serum, SERS, PCA

## Abstract

Shiga toxins-producing *Escherichia coli* (STEC) are zoonotic pathogens causing severe diseases such as hemorrhagic colitis (HC) and hemolytic uremic syndrome (HUS). Infections caused by STEC represent a public health concern due to the severity of the possible outcome and acute mortality. The early diagnosis of the infection is pivotal to driving a correct therapeutic protocol to limit the severity of the symptoms. The diagnosis is quite cumbersome, requires specialized approaches, and thus is rarely performed in the hospital, being managed by the relevant national reference laboratory, delaying the administration of the appropriate supportive care. In this context, the demand for affordable diagnostic tests to be carried out at the bedside is crucial for providing high-value healthcare. In this study, for the first time to the best of our knowledge, we developed and optimized a highly sensitive SERS-based platform that can detect and identify the two main Shiga toxin variants (Stx1 and Stx2a) as well as the cleaved form of Stx2a in human blood serum at extremely low concentrations with limits of detection reaching 0.007 ng/mL (0.1 pM). This method uses affordable, sensitive, and very efficient SERS substrates based on gold nanoparticle films, made with a cost-effective bottom-up approach, which are much cheaper than those typically found in the literature. Our results show that the platform works well in complex biological samples, offering high sensitivity and specificity. Moreover, integrating machine learning algorithms, such as principal component analysis (PCA), enables accurate identification of toxin types, overcoming the limitations of conventional diagnostic methods. This innovative approach represents a significant step toward accessible, rapid, and scalable clinical diagnostics, potentially transforming the early detection and management of STEC-related infections and preventing life-threatening complications.

## Graphical abstract

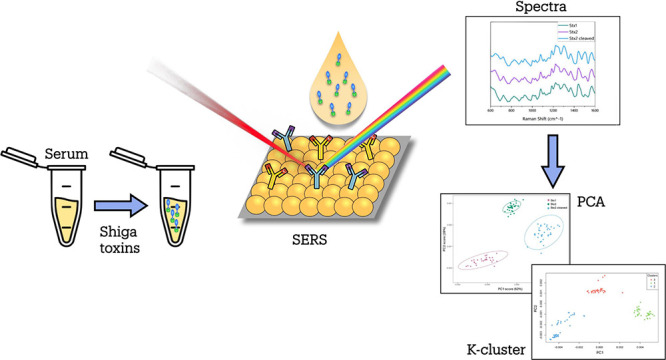

## 1 Introduction

Accurately detecting and differentiating the main Shiga toxin (Stx) types, Stx1 Stx2, and the cleaved form of the latter (cleaved Stx2) is a critical challenge in clinical diagnostics and public health^1–6^ These toxins, produced by certain pathogenic strains of *Escherichia coli* (Shiga toxin-producing *E. coli*; STEC), are among the most potent bacterial toxins implicated in human disease. Structurally, Stxs consist of two components: an A subunit, responsible for the enzymatic activity, and a pentamer of the B subunit, which facilitates binding to host cell receptors (globotriaosylceramide, Gb3). The toxin’s mechanism of action starts with the B subunit binding to host cell surfaces, allowing the A subunit to enter the cytoplasm. Once internalized, the A subunit halts protein synthesis by cleaving a specific adenine residue from the 28S rRNA, leading to cell death.^7^ Stx are categorized into two major antigenic types, Stx1 and Stx2. Stx2, particularly its subtypes (e.g., Stx2a, Stx2c, and Stx2d), is strongly associated with the most harmful consequence of STEC infections in humans, namely hemolytic uremic syndrome (HUS), while Stx1 typically is displayed by strains causing diarrhea (sometimes bloody) due to intestinal cell damage, abdominal pain resulting from inflammation, fever, and dehydration from fluid loss. Stx1 is rarely linked to HUS, but in rare cases, it may lead to complications like anemia, low platelets, and kidney failure, especially with additional risk factors.^8^

Epidemiological data show that the risk of developing HUS is highest in infections caused by STEC producing Stx2, while dual production of Stx1 and Stx2 reduces the likelihood of severe disease. HUS primarily affects young children but can occur at any age, often following ingestion of food or water contaminated with STEC. The intimate adhesion of these bacteria to the mucosa of the bowel causes diarrhea, followed by the release in circulation of the potent Stxs. The toxins target suddenly the endothelial cells of the gut leading to bloody diarrhea, then bind to several circulating blood cells (monocytes, neutrophils, and platelets). These cells are stimulated by toxin binding and release large extracellular vesicles which deliver a toxic cargo (Stx and other virulence factors) to the glomerular endothelial cells of the kidney. HUS arises as Stx-induced damage to endothelial cells in the kidneys triggers platelet activation and promotes the formation of microthrombi. This cascade results in the hallmark features of HUS: microangiopathic hemolytic anemia, thrombocytopenia, and acute renal failure. Effective management of HUS requires prompt diagnosis, supported by clinical presentation, laboratory findings, and microbiological assays. Treatment focuses on supportive care, including fluid management, blood transfusions, and dialysis. Long-term complications, such as chronic kidney disease, hypertension, and neurological issues, underscore the importance of early intervention.^5^

Recent research^6,8,9^ highlighted the circulation in the patient’s serum of a cleaved form of Stx2a, in which the A subunit splits into two fragments (A1 and A2) connected by a disulfide bond. This cleaved variant exhibits different binding properties for human circulating cells compared to the native toxin, contributing differently to disease progression. The appearance of the cleaved Stx2a variant is less understood but is suspected to play a critical role in pathogenesis, warranting further investigation.

Conventional diagnostic methods, including culture techniques, ELISA, and PCR, fall short in meeting the demands of rapid and accurate toxin identification and characterization, particularly when it comes to distinguishing between closely related variants in complex biological matrices like human serum. These methods often suffer from lengthy processing times, limited sensitivity, and poor multiplexing capabilities. The complex composition of serum (with its array of proteins, lipids, and biomolecules) further complicates detection, increasing background noise and reducing specificity.

This diagnostic bottleneck highlights the pressing need for innovative tools capable of delivering high accuracy, sensitivity, and reproducibility in toxin detection.^10–13^

Recent advancements in biosensing technologies^10–15^ particularly those based on plasmonic nanostructures, offer transformative solutions.^16,17^ Surface-Enhanced Raman Spectroscopy (SERS) has gained attention for its sensitivity and ability to generate unique molecular fingerprints through the amplification of Raman signals.^18^ This is achieved by exploiting the localized surface plasmon resonance properties of metallic nanostructures, such as gold and silver nanoparticles.^19–22^ In this context, SERS platforms based on plasmonic nanostructures, such as nanoparticles, present a powerful alternative. SERS achieves remarkable signal amplification primarily through electromagnetic mechanisms driven by localized surface plasmon resonances (LSPRs).^23^ These resonances arise when light interacts with metallic nanostructures, inducing collective oscillations of conduction electrons at the nanoparticle surface. When nanoparticles are closely spaced, strong plasmonic coupling can occur, resulting in the formation of highly localized electromagnetic “hot spots” in the narrow interparticle gaps.^24^ Molecules located in these regions experience intense field enhancement, often boosting Raman signals by several orders of magnitude.^25^

Several studies have demonstrated the potential of SERS for detecting bacteria, viruses, and toxins, underscoring the importance of substrate design in achieving high performance. Indeed, these nanostructures, precisely engineered to enhance local electromagnetic fields, significantly boost the detection process.^26–30^ In systems based on Langmuir–Blodgett (LB) self-assembly, gold nanoparticles (Au NPs) are organized into highly ordered and densely packed monolayers with controlled interparticle distances. This structural regularity ensures consistent generation of hot spots across the substrate, optimizing both the intensity and reproducibility of the SERS signal.^31^ Additionally, the ordered arrays can support collective plasmonic modes, enhancing uniformity over large areas. These combined effects make LB-fabricated films a powerful platform for developing sensitive and reproducible SERS-based sensors.^32^ This intense and localized electromagnetic enhancement plays a central role in enabling the extremely low detection limits achieved with our SERS substrate, as it significantly boosts the Raman signal even from trace amounts of analyte.^33^

AuNPs are among the most widely used plasmonic materials for SERS due to their chemical stability and strong localized surface plasmon resonance.^34^ While colloidal AuNPs are commonly employed in suspension for rapid SERS analysis, their stochastic aggregation and lack of structural control often result in poor signal reproducibility and inconsistent enhancement.^35^ In contrast, the approach based on LB self-assembly enables the fabrication of highly ordered monolayers with uniform interparticle spacing on solid substrates. This controlled architecture ensures a dense and reproducible distribution of electromagnetic hot spots and improving substrate stability and batch-to-batch consistency. Compared to conventional colloidal systems, the self-assembled Au monolayer provides a more robust and tunable platform, which is particularly advantageous for applications requiring high sensitivity and reliable spectral performance.^31,32,36,37^ The fabricated nanostructures, precisely engineered to enhance local electromagnetic fields, significantly boost the detection process.^26,28,30,38^

By optimizing parameters such as size, shape, and arrangement, it becomes possible to detect specific toxins even at trace levels among the complex environment of human serum.^29,30^ Furthermore, SERS is a label-free technique, eliminating the need for external labels or colorants that complicate assay preparation and introduce variability. SERS stands out as a promising candidate for detecting Stxs in human serum. It allows clear differentiation between variants such as Stx1, Stx2a, and cleaved Stx2a based on their distinct vibrational patterns. Moreover, advanced analytical methods, such as Principal Component Analysis (PCA), further enhance SERS capabilities by enabling the precise classification of toxin variants based on fine spectral differences.^39^

In this study, we present a novel cost-effective SERS-based diagnostic platform for real-life applications of SERS utilizing gold nanoparticles substrates for the specific detection of Stx1, Stx2a, and cleaved Stx2a in human serum. By combining the molecular specificity of Raman spectroscopy with the signal enhancement of our biosensors, fabricated using very simple and low-cost methods, this platform achieves label-free and ultrasensitive detection of Stxs. Our PCA results, used to analyze small differences in the SERS spectra, show that combining SERS with PCA can be an effective and fast method for detecting and typing Stx without requiring probes or labels demonstrating the sensor’s ability to overcome the limitations of conventional methods, offering a rapid, reliable, and scalable solution. Our results can potentially revolutionize diagnostics for STEC infections, enabling timely clinical management and reducing the incidence of severe complications like HUS. This approach connects advanced nanotechnology with real-world clinical needs, opening the door to easy-to-use, point-of-care diagnostic tools that address one of the biggest challenges in detecting infectious diseases.

## 2 Materials and Methods

### 2.1 Chemicals

Hydrogen tetrachloroaurate (III) trihydrate (HAuCl_4_·3H_2_O), trisodium citrate (Na_3_C_6_H_5_O_7_·2H_2_O, 99%), and toluene were purchased from Carlo Erba Reagents. 4-Mercaptobenzoic acid (4MBA, 99%), phosphate-buffered saline (PBS, pH 7.4, liquid), and p-type Si (100) wafers (boron-doped, resistivity 0.7–1.3 Ω·cm) were obtained from Sigma-Aldrich. Sulfuric acid (H_2_SO_4_, 90–98%) was sourced from Alfa Aesar, while hydrogen peroxide (H_2_O_2_, 30%) came from VWR Chemicals. Bovine serum albumin (BSA) was obtained from Biowest.

### 2.2 Treatment of the Silicon Wafer

A silicon wafer was cleaned by immersing it in a solution of H2SO4:H2O2 (7:3 by volume) at room temperature for 2 h, following a typical hydrophilic treatment. It was then thoroughly rinsed with deionized water and dried using nitrogen gas. The sample is ready for activation using the Diener Pico plasma cleaner under low pressure at room temperature, operating at 80% power for 60 s.

### 2.3 Synthesis of Gold Nanoparticles (Au NPs)

Au NPs stabilized with citrate were synthesized by reducing tetrachloroauric acid. To create a wine-red solution, 1 mL of 1% sodium citrate was added to 100 mL of a boiling solution of HAuCl4 (10^–4^ g/mL) while stirring, and the mixture was boiled for 10 min.^40,41^ While continuing to boil, 1 mL of the sodium citrate solution (1%) and 1 mL of the HAuCl4 solution (10^–4^ g/mL) were added every 2 min, repeating this process three times. The solution was then heated at 100 °C for an additional 20 min before being left to cool naturally. At the end of the reaction, 24 mL of the nanoparticle solution was centrifuged at 8000 rpm for 10 min to collect the precipitate, which was then redispersed in 50:50 deionized water–ethanol solution for the formation of the Au NPs layer.^37^

### 2.4 SERS Substrate Fabrication

The SERS substrate was fabricated by assembling Au NPs at the interface between two immiscible liquids, following a modified method.^37^ First, 8 mL of toluene was carefully poured on top of 80 mL of water to form a stable liquid/liquid interface. Next, 4 mL of water–ethanol dispersed Au NPs was slowly introduced to the interface using a mechanical syringe pump at a flow rate of 3 mL/h. As the toluene evaporated, the Au NPs naturally self-assembled into a monolayer at the liquid interface. Finally, this monolayer was transferred onto a silicon wafer.^39^

### 2.5 SEM Characterization

The nanostructures fabricated were characterized morphologically by making use of scanning electron microscopy (SEM, Raith 150).

### 2.6 Reflectance Spectroscopy: LSPR Measurement

LSPR reflectance spectroscopy was employed to investigate the plasmonic characteristics of the sensor using a home-built optical setup. A broadband white light laser source was directed onto the self-assembled monolayer of gold nanoparticles deposited on a silicon substrate. The light reflected from the sensor surface was captured through a fiber-optic cable and subsequently analyzed using a USB4000 spectrometer. The reflectance spectra were recorded across the 400–1050 nm wavelength range. Additional details of the optical arrangement are provided in the Supporting Information (SI), Figure S1.

### 2.7 SERS Measurement

SERS spectra were recorded using a Raman system (QE Pro-Raman, Ocean Optics) coupled with an upright microscope (Olympus BX51) in a backscattering configuration. The system was configured for λ = 785 nm with a grating of 1200 lines/mm and an input slit of 50 μm. Spectra were collected using a 50× (N.A. = 0.75) objective and a laser power of 12 mW. In the case of Stx sensing, for each sample, 40 SERS spectra from different points were collected in the range of 350–1400 cm^–1^ with an acquisition time of 10 s. In all cases investigated, spectra recorded were subsequently baseline corrected and averaged using the software Omnic.

### 2.8 SERS Toxins Detection

#### 2.8.1 Toxins and Antibodies

Stx1a and Stx2a were produced by *E. coli* C600 (H19J or 933W, respectively) and purified by receptor analog affinity chromatography. Both toxins were further purified on ActiCleanEtox columns (SterogeneBioseparations, Carlsbad, CA, USA) to remove contaminant bacterial endotoxin. Cleaved Stx2a^6^ was obtained by incubating native Stx2a (120 μg) with trypsin (1 μg) in 100 μL-PBS (1 h at 37 °C) followed by the addition of phenylmethylsulfonyl fluoride (14 ng) as inhibitor (10 min at 37 °C, final reaction volume 120 μL). Purified toxins were stored at −80 °C in small aliquots and diluted with PBS before each assay. The functionalization of the proposed platforms was achieved by using monoclonal antibodies (Toxin Technology, Sarasota, Florida, United States) to Stx1 (Stx1–13C4) and Stx2 (Stx2-BB12).

#### 2.8.2 Fingerprint Analysis

For the fingerprinting of the toxins, droplets of 1.047 × 10^4^ ng/mL solutions of each toxin were directly deposited on the substrates. Separate solutions were prepared in PBS and human serum at 1.047 × 10^4^ ng/mL. The PBS solutions were used to obtain representative SERS peaks specific to each toxin, while the solutions in human serum allowed for the identification of toxin peaks that remain detectable within the complex serum matrix. A droplet of pure human serum was also deposited under identical conditions to serve as a control. Following droplet deposition, the substrates were incubated overnight in a humid chamber at 4 °C. The samples were washed seven times with 1 mL of bidistilled water and dried with N_2_ gas before SERS spectral data collection.

#### 2.8.3 Monoclonal Antibodies Physioadsorption

The proposed biosensor was constructed by noncovalently functionalizing the nanopatterned gold surface through the physisorption of monoclonal antibodies specific to Stxs (Figure 1). Specifically, 50 μL of a 10 μg/mL monoclonal antibody solution in PBS was applied dropwise onto the nanostructured chip surface. The chip was then placed in a humid chamber at 4 °C overnight, allowing for the physical adsorption of antibodies targeting Stx1 (AbStx1) and Stx2a (AbStx2, also for cleaved Stx2a) onto the gold surface. Following incubation, the chip was thoroughly washed five times with 1 mL of bidistilled water to eliminate any unbound antibodies.

**Figure 1:**
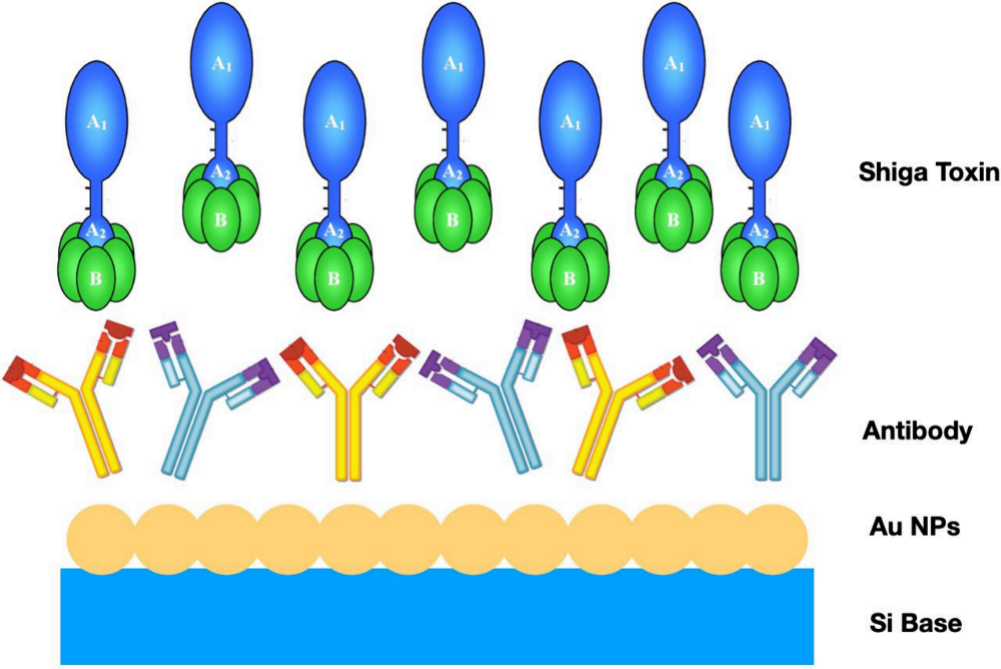
Schematic representation of the attachment of antibodies to the nanobiosensor surface via physical adsorption.

To minimize nonspecific signals, a blocking step was performed before toxin deposition. The sensor surface was passivated with a 1% w/w BSA solution, incubated for 1 h at 25 °C in a humid chamber, and then rinsed with bidistilled water. A toxin solution with a final concentration of 0.1 ng/mL in human serum was then applied to the biofunctionalized nanostructures to evaluate biosensor performance. The solution was incubated on the chip overnight at 4 °C in a humid chamber. After incubation, the sensor was again washed five times with 1 mL of bidistilled water and dried with N_2_ before SERS spectrum acquisition.

#### 2.8.4 Limit of Detection - LOD

We used the same functionalization procedure to evaluate our system’s limit of detection (LOD). We performed repeated measures of toxin solution, at concentrations of 0.0015–1.5 nM. The nanosensor was incubated with a toxin solution for each measurement, and the SERS peak was measured after washing.

### 2.9 Data Analysis

The spectra were analyzed using the Origin2023 software. Baseline corrections and spectral normalization were performed to ensure accurate comparison across samples. Peak assignments were also conducted within Origin2023 to facilitate the consistent identification of characteristic toxin peaks.

PCA is a statistical technique for reducing the dimensionality of data sets while preserving the most important variance. In this study, PCA was employed to discriminate between Stx1, Stx2a, and cleaved Stx2a. Their SERS spectra are nearly identical, making visual differentiation extremely difficult. This distinction is of great clinical importance for accurate toxin identification. The analysis was performed using R version 2024.04.2 + 764 and the ChemoSpec package.

Following PCA, a k-cluster analysis was conducted using the cluster package in R to categorize the samples into distinct groups based on similarities in the first two principal components (PC 1 and PC 2). The k-means clustering method was chosen to provide an initial partitioning, allowing us to explore whether the spectral differences could effectively group the toxin types.^42^ Each cluster was then assigned a label corresponding to the toxin type, and the clustering results were reviewed for coherence and separability. We chose k-means clustering as a simple and intuitive unsupervised learning method that complements PCA, which is also unsupervised. This approach allowed us to assess whether the classes are intrinsically separable without relying on labeled data or supervised training.

After the k-cluster analysis, an analysis of variance (ANOVA) was applied to PC 1 and PC 2 using the stats package in R to determine if significant differences existed between the toxin groups identified. Specifically, a one-way ANOVA was performed on each principal component independently to evaluate the effect of toxin type on the spectral features extracted by PCA.

### 2.10 Preliminary Assessment on HUS Patient Serum Samples

To preliminarily assess the applicability of our SERS platform to real clinical samples, we analyzed human serum obtained from four patients diagnosed with HUS in the early and late stages of the disease. The samples have been provided by coauthor Dr Ardissino from the Center for HUS Control, Prevention and Management (Ospedale Maggiore Policlinico, Milan). These sample were previously tested with RT-PCR to assess the presence of *stx1* and/or *stx2* genes in feces.

Based on the RT-PCR results, one of the two samples collected early was confirmed to contain both Stx1 and Stx2, while the other contained only Stx2. The samples collected in the late stage of the disease were expected to contain negligible concentrations of Stxs and, accordingly, the result of the assay was negative. Four separate assays were conducted using anti-Stx2 and anti-Stx1 antibodies, respectively.

For each toxin, SERS substrates were functionalized with the corresponding antibody and incubated with (i) the patient serum as received, and (ii) the same serum spiked with 5 ng/mL of the target toxin, used as a positive control. The spiked concentration was selected to ensure a measurable signal while remaining close to the clinically relevant range. Spectral acquisition was performed as described in previous sections, and PCA was used to evaluate the similarity between spiked and unspiked samples.

## 3 Results and Discussion

In this work, we tackle the challenge of creating a SERS-based detection platform capable of rapid, in situ analysis of Stxs, potent cytotoxins implicated in severe human diseases. We developed a gold-based plasmonic substrate engineered for optimal signal enhancement and specificity to achieve this. Gold-based SERS substrates were developed using monodisperse Au NPs assembled into large-scale monolayers via a liquid–liquid self-assembly method leveraging the Marangoni effect. These substrates exhibit uniform nanoparticle distribution and minimal defects, optimizing local electromagnetic field enhancement for Raman signal amplification. The plasmonic substrate was optimized to operate with high near-field enhancement at 785 nm, the laser source wavelength of our SERS system. This was achieved by carefully calibrating structural parameters, which enhance resonance and field localization.

Our substrate enables the detection and precise discrimination of Stx variants—Stx1, Stx2a, and cleaved Stx2a— directly in human serum, addressing a significant challenge in clinical diagnostics. Leveraging PCA as the primary tool for data clustering, we achieved robust and reproducible discrimination of the three toxin forms, even in complex biological matrices. This was further reinforced through validation by ANOVA and k-means clustering, confirming the reliability and accuracy of our analytical approach.

Notably, the system exhibits an exceptionally low LOD of 0.007 ng/mL in serum, making it an ideal tool for early stage detection where timely intervention is critical. The unparalleled specificity of our platform is attributed to the use of highly selective monoclonal antibodies targeting Stx1 and Stx2a, which ensure precise recognition while minimizing the risk of cross-reactivity and false positives. This substrate, consisting of self-assembled gold nanoparticles, has already been studied and characterized by us in a previous work,^39^ including its temporal stability, measurement repeatability, and reproducibility. The sensor exhibits an enhancement factor of approximately 10^7^; further details on the calculation and mechanism are provided in the SI.

Importantly, this high-performance substrate also exhibits a favorable cost profile. The total fabrication cost, including materials, processing, and personnel time, is approximately €15 per unit, which is significantly lower than many commercially available SERS substrates. A detailed cost breakdown and comparison are also provided in the SI.

The substrate’s stability has been previously assessed. Its shelf life is up to three months, and full details are provided in our earlier publication.^39^

Finally, to further evaluate the clinical relevance of the platform, we analyzed serum samples collected from four patients with confirmed STEC infections. One sample contained only Stx2, while the other included both Stx1 and Stx2. Despite the extremely low concentrations of circulating toxin, consistent with the rapid clearance of Stxs from the human body, we were able to detect and correctly classify the presence and subtype(s) of the toxins using our SERS platform. These findings demonstrate the system’s practical utility in real-world scenarios and its potential as a diagnostic aid for Stx-related diseases.

This work represents a transformative step for clinical diagnostics, offering a powerful tool for accurate toxin profiling. By enabling both high sensitivity and specificity in a user-friendly SERS- based platform, this study lays the foundation for real-world applications in managing toxin-related diseases.

### 3.1 SERS Substrate Characterization

Monodisperse Au NPs were made using the sodium citrate reduction method. Then, a large-area monolayer film of Au NPs was created through a liquid–liquid self-assembly process based on the Marangoni effect (Figure 2b).^37,40–42^ A SEM image of the fabricated Au NPs layer is presented in Figure 2a. The Au NPs films were uniformly self-assembled, with no significant clustering or large gaps. This uniformity is crucial for enhancing local electromagnetic fields, which significantly amplify the Raman signal and improve the detection process. Based on the SEM analysis, the size distribution of the Au NPs is monodispersed, with a mean size of 43.3 ± 8.7 nm.

**Figure 2:**
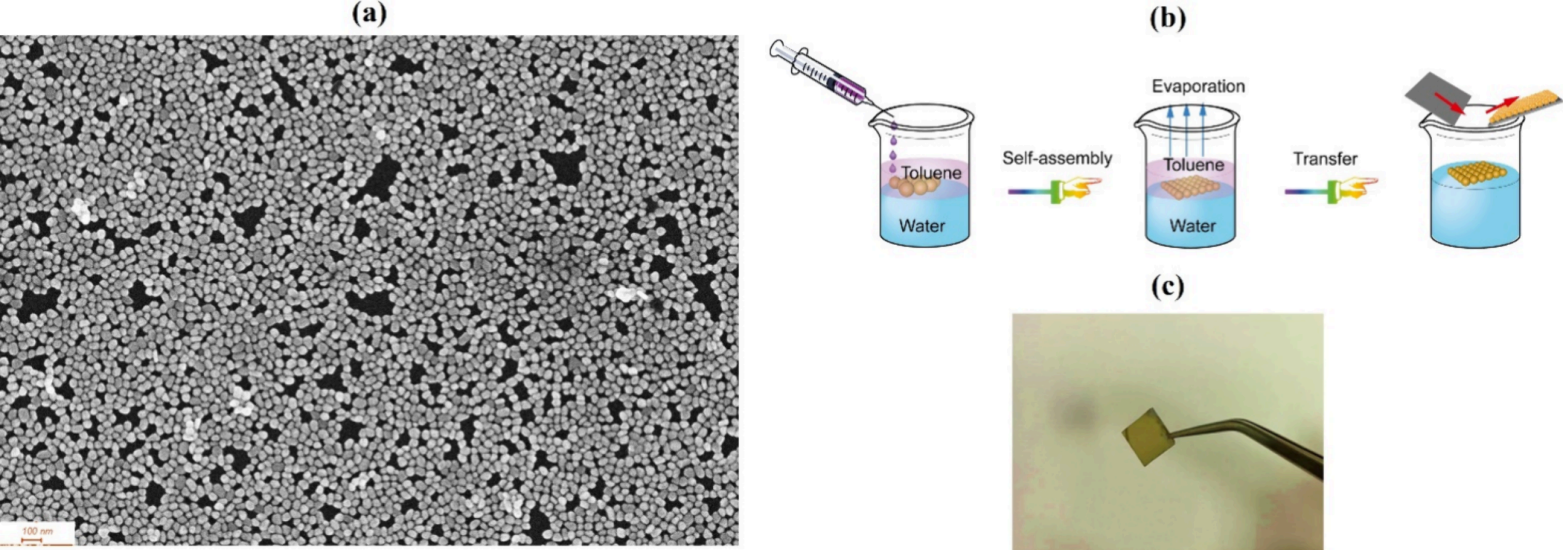
(a) Scanning electron microscopy (SEM) image - scale bar: 100 nm of the fabricated Au NPs layer. (b) Fabrication procedure to obtain gold nanoparticles self-assembled monolayer (c) Photograph of the SERS substrate.

Gold nanoparticles with an average diameter of 43.3 ± 8.7 nm were selected as they offer a favorable balance between strong plasmonic resonance and effective electromagnetic field confinement. In this size range, the extinction cross-section is maximized near the excitation wavelength (785 nm), enhancing the localized surface plasmon resonance and thus the SERS signal. Although larger gold nanoparticles (e.g., 60 nm in diameter) have been shown in simulation studies to generate stronger local electric fields due to multipolar plasmon modes, they also exhibit broader extinction peaks and red-shifted resonance positions that may reduce coupling efficiency with the excitation laser (785 nm in our case). In contrast, the chosen size offers a well-defined localized surface plasmon resonance, balanced field enhancement, and better compatibility with the excitation wavelength, ensuring both high SERS performance and reproducibility.^43–46^

The responsiveness of AuNP monolayers via LSPR reflectance spectroscopy serves as a predictive tool for SERS performance. LSPR reflectance spectroscopy measures the resonance wavelength where collective oscillation of conduction electrons occurs due to incident light. SERS intensity depends strongly on the local electric field enhancement, which is maximized when the excitation wavelength is close to the LSPR peak of the AuNP monolayer. Maximum SERS enhancement is typically achieved when: (i) the LSPR peak is resonant with the excitation laser; (ii) there is strong interparticle coupling, forming numerous hot spots; (iii) there is spectral overlap between LSPR and both excitation and Raman-scattered light. The reflectance spectra of our sensor obtained by LSPR reflectance spectroscopy, is reported in SI as Figure S2. The maximum LSPR peak is at 640 nm. From the experimental results we have a very good overlapping with the excitation wavelength, explaining the high SERS performance.

### 3.2 Substrates’ Enhancement Factor

The SERS performances of the fabricated platform have been reported in the SI and introduced in our earlier work (Zhou et al., 2023).^39^ The self-assembled AuNPs fabricated film was analyzed using 4MBA as a Raman probe. The SERS spectrum of 4MBA (Figure S4 in the SI) displays well-defined fingerprint peaks, with a prominent band at 1079 cm^–1^ corresponding to in-plane ring breathing and C–S stretching vibrations, and another at 1588 cm^–1^ attributed to C–C stretching of the aromatic ring.^16,26,39^ To estimate the SERS Enhancement Factor (EF), we compared the SERS spectrum of 4MBA with its conventional Raman spectrum acquired on a silicon wafer. The substrates exhibit an enhancement factor of 3.13 × 10^7^, a value that is crucial for achieving strong signal amplification and enabling highly sensitive detection of low-concentration analytes.

### 3.3 Toxins Analysis in Real Samples by Unfunctionalized SERS Substrates: Toxins Fingerprints

The plasmonic metasurface was evaluated for its ability to detect and characterize the SERS fingerprint spectra of the three Stxs types under study. Each toxin was deposited on the same substrate at a concentration of 1.047 × 10^4^ ng/mL, prepared separately in PBS and human serum. We took 40 measurements for each toxin droplet, yielding repeatable spectra with well-defined peaks. Figures 3a,b display the average SERS spectra obtained in PBS and human serum, respectively, for Stx1 (pink line), Stx2a (green line), and cleaved Stx2a (light blue line). In Figure 3b, the human serum spectrum is also shown in black.

**Figure 3:**
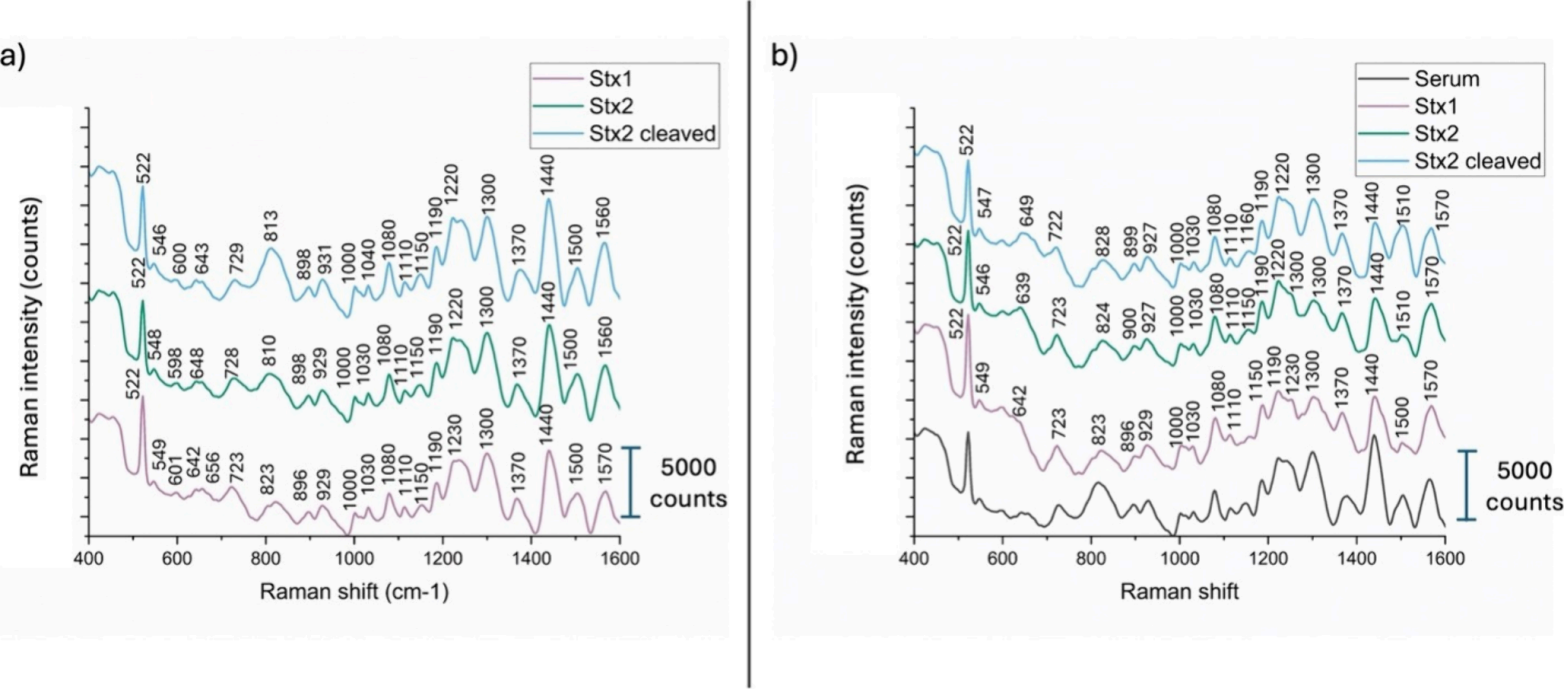
SERS spectra of the three toxins measured in (a) PBS and (b) serum. The spectra in panel (a) show the characteristic signals of the three toxins in PBS (Stx1 as pink curve, Stx2a as green curve, cleaved Stx2a as light blue curve) while panel (b) presents their respective spectra in serum alongside the spectrum of serum alone (black line).

The apparent similarity between the SERS spectra of serum (Figure 3b) and of the Stx variants (Figure 3a) is because both are protein-based systems. Human serum is rich in proteins (e.g., albumin, globulins), and the Shiga toxins are themselves proteins composed of the same 20 standard amino acids. As a result, their SERS spectra share many common vibrational bands (e.g., from amide groups, aromatic residues, C–H and N–H stretching), which gives rise to overall similar spectral profiles. However, despite these similarities, PCA and other multivariate techniques reveal distinct clustering of each toxin, indicating that subtle spectral differences—likely arising from variations in secondary/tertiary structure and amino acid composition—are captured and can be reliably used for discrimination.

The spectra of the toxins in serum exhibit some visible differences compared to those in PBS. For instance, the peak at 810 cm^–1^ shifts for Stx2a and cleaved Stx2a to 824 cm^–1^ (Tyr Fermi resonance) for the toxins in serum, the peak at 1303 cm^–1^ (Amide III vibration corresponding to α-helices) is less pronounced for Stx2a and Stx1, the peak at 1440 cm^–1^ (stretching CH) is also less pronounced for Stx2a and cleaved Stx2a, the peak at 1510 cm^–1^ (Amide I vibration) comes out for cleaved Stx2a and vibration at 1500 cm^–1^ is blunted in Stx1 and Stx2a in the presence of serum. Overall, the human serum spectrum (black line in Figure 3b) closely resembles both the spectra of the toxins in PBS and those of the toxins in serum, due to the protein-rich composition of serum. However, differences in the relative intensities of specific peaks allow for the differentiation of toxin spectra from that of serum. Notably, the peak intensity ratios among the three toxins in serum are more similar, with only subtle distinctions, than those of serum alone.

The SERS analysis of Stx1, Stx2a, and cleaved Stx2a in serum revealed distinct spectral fingerprints that highlight the toxins’ unique structural and chemical characteristics. Table 1 provides a full SERS band assignment. Through comparative analysis of peak assignments and intensities, significant differences emerged that emphasize the impact of molecular variations and cleavage on their vibrational properties.

**Table 1:** Assignment of the Main Peaks Observed in the SERS Spectra of the Toxins in Serum^a^

Wavenumber (cm^–1^)	Assignment	Stx1	Stx2	Stx2 cleaved
639	Tyr ring deformation^29^		+	
649	Tyr, ν(C–S)^47^			+
722	Tyr^48^ Ile^49^ met C–S stretching;^29^ v(C S)T^50^	+	+	+
824	Tyr^51^ Fermi resonance between ring breathing and out of plane ring bend overtone^48^	+	+	+
900	CS bond or aromatic ring^52^ Trp^47^	+	+	+
927	Ring breath vibration of C–O^52^ Proline: ring ν(CC)^51^ Ile ν(COO−)^49^ ν(CC)^53^	+	+	+
1000	Symmetric ring breathing mode of phenylalanine^48^ Symmetric CC ring stretching^48^	+	+	+
1030	C–H stretching mode of phenylalanine^54^	+	+	+
1080	Strong C9 H’s stretch^52^ causing a strong inner ring distortion on C9, C10, C11, wagging of H39, asymmetric stretch of benzene ring at C11, wagging of H40 ^52^ n(Ca–N)^55^ Trp^48^	+	+	+
1120	ν(CN)^47^	+	+	+
	Stretching (CN) protein^29^			
1160	Tyr^49^ In-plane CH bending, ring CH bending^48^	+	+	+
1190	Phe^51^ Combination of in-plane CH bending^48^ Tyr^47^	+	+	+
1220	Phenyl-C stretching^47^	+	+	+
1303	CH2 wag or Ring stretching^47^ Amide III α helix^29^	+	+	+
1373	δ (COO), v_s_ (COO−)^53^	+	+	+
1440	stretching C–H^56^	+	+	+
1510	Vibrational mode of the amide^52^		+	+
1570	Trp^48^	+	+	+

a The table lists the characteristic vibrational modes and their corresponding wavenumbers, providing a detailed interpretation of the spectral features.

Interestingly, there are few differences in the vibrational modes identified among the three toxins. First, Tyr peak in the region 639–649 is absent in Stx1 and unique and different for Stx2a (at 639 cm^1^) and for cleaved Stx2a (at 649 cm^–1^) highlighting differences between wild-type Stx2a and its cleaved variant. Another notable difference among the two Stx2a and Stx1 that emerges from these spectra is in the vibrational regions corresponding to the α-helix conformation whose main peaks (at 1300 cm^–1^ and 1510 cm^–1^) are progressively more pronounced in the following order, cleaved Stx2a > Stx2a > Stx1). The remaining peaks suggest that certain conformational features are conserved across all three toxins.

Given that the spectra of the three toxins are highly similar, especially in serum, and that the characteristic peaks of each toxin — although consistently present — shift by a few cm^–1^ across different experimental replicates, it can be challenging to determine whether the spectrum corresponds to a toxin in serum, and, if so, which one of the three it is. To address this, we employ PCA to discriminate the three toxins effectively. As shown in Figure 4, PCA enables us to reduce the dimensionality of the spectral data, highlighting the subtle differences between the toxins and allowing for a more accurate and intuitive classification. By transforming the data into principal components, we can visualize and distinguish the toxins more reliably and easily to interpret, even for those less familiar with the intricacies of spectroscopic analysis.

**Figure 4:**
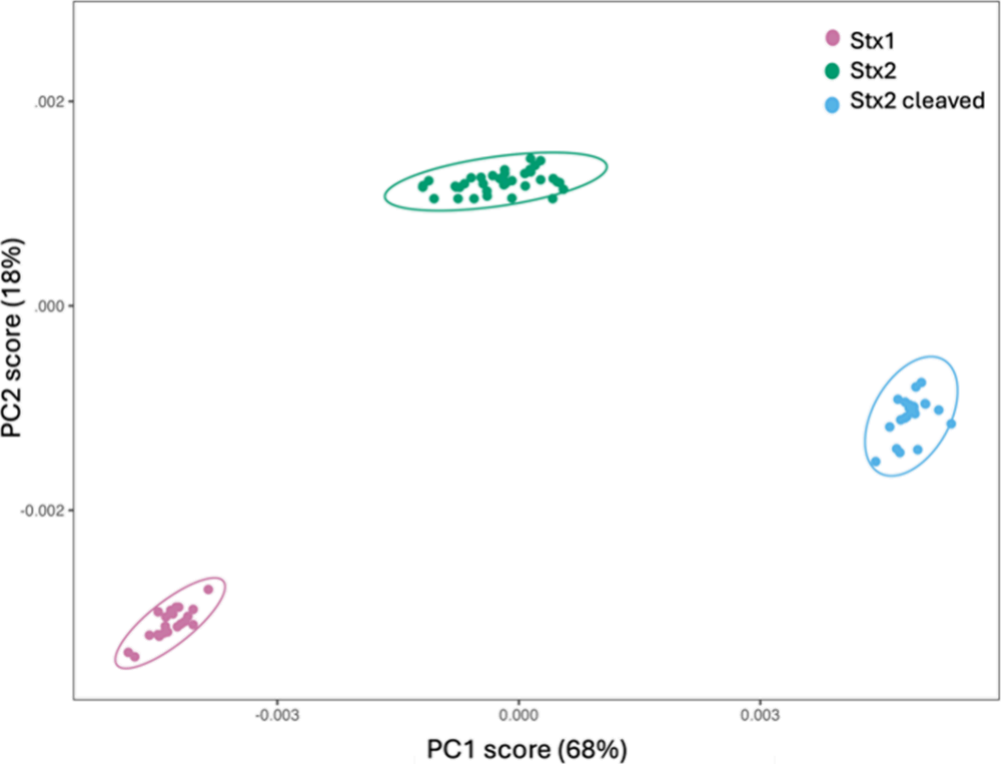
PCA of the SERS spectra for the three toxins in serum (Stx1 as pink dots, Stx2a as green dots, cleaved Stx2a as light blue dots).

Figure 4 presents the distribution of data points in a 2D space defined by the first two principal components (PC1 and PC2), representing Stx1 (pink dots), Stx2a (green dots), and cleaved Stx2a (light blue dots) based on SERS measurements. The map separates the clusters corresponding to each toxin, indicating successful discrimination. This separation is supported by the high explanatory power of the principal components, with PC1 accounting for 68% of the variance and PC2 for 18%, surpassing the 80% threshold typically considered adequate for reliable analyte differentiation. These findings underscore that SERS, combined with PCA, provides an effective approach for detecting and distinguishing Stx variants in human serum, enabling specific analyte recognition without reliance on antibody probes.

To validate the PCA model’s ability to discriminate among the three toxins, we applied k-cluster analysis in conjunction with ANOVA testing.^42^ K-cluster analysis is a statistical technique used to partition data into k distinct clusters based on similarity, minimizing variance within each cluster while maximizing the separation between clusters. In this study, it was used to classify the spectral data into three distinct clusters, each corresponding to one of the toxins. This approach enabled us to visually and quantitatively assess whether the PCA-transformed data could reliably differentiate between the toxin types.

As shown in Figure 5, the k-cluster analysis effectively grouped the spectra of each toxin type into unique clusters. Specifically, all spectra for Stx1 were consistently assigned to one cluster, while Stx2a and cleaved Stx2a spectra were similarly grouped into their respective clusters, as shown in Table 2. The clustering results show a precise separation where each toxin type forms a distinct group with no overlap, indicating a clear discrimination among the toxins based on their spectral signatures.

**Figure 5:**
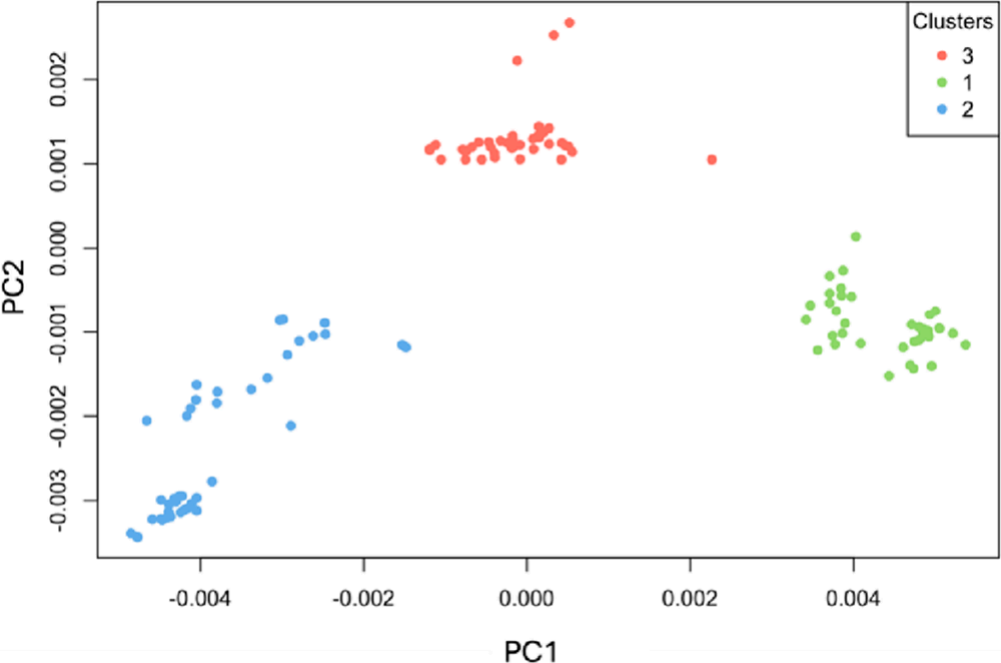
Cluster analysis of PCA-transformed spectra for toxin discrimination.

**Table 2:** Cluster Assignment of Toxin Spectra

	Stx1	Stx2	Cleaved Stx2
1	0	40	1
2	0	0	39
3	40	0	0

To statistically confirm the effectiveness of this discrimination, we also conducted an ANOVA test, which showed a highly significant separation among the groups (F^2, 117^ = 355.6, *p* < 2e^–16^). This result underscores the robustness of the PCA model in differentiating between toxins, even when spectral similarities might otherwise obscure such distinctions. Combining PCA with k-cluster analysis and ANOVA test thus provides a reliable framework for toxin classification, facilitating precise identification even under complex spectral conditions. We attribute the clear separation observed in the PCA plot to two key factors. First, as previously noted, the high-quality and uniform SERS signals provided by our self-assembled substrate enable the detection of subtle spectral variations with excellent reproducibility. This allows for precise representation of molecular differences in the Raman fingerprint. The gold nanoparticle monolayer was produced via LB self-assembly, a method that ensures high structural regularity, dense nanoparticle packing, and reproducible interparticle distances. These characteristics promote consistent hot spot formation across the surface, which is critical for both high enhancement and signal uniformity.

We believe that the excellent performance observed in the PCA plots, characterized by 100% classification accuracy and no spectral overlap, is the result of the combined effect of high SERS enhancement and exceptional signal uniformity. This synergy enables not only strong Raman signal amplification but also the preservation of subtle spectral differences between closely related analytes. As a result, the spectral data retain the molecular specificity necessary for clear clustering and effective discrimination.

Moreover, although Stx1, Stx2, and cleaved Stx2 all belong to the Shiga toxin family, they present notable structural differences. Stx1 and Stx2 share only ∼ 50% sequence similarity, while cleaved Stx2, despite originating from Stx2, differs in its folding and conformational properties. These variations translate into distinct vibrational fingerprints that can be readily captured by SERS and resolved by unsupervised PCA, without the need for more complex classification algorithms.

### 3.4 Toxins Analysis in Real Samples by Functionalized SERS Substrates with Physically Adsorbed Antibodies

A second approach was explored using functionalized SERS substrates to develop a sensor capable of detecting and discriminating among the three Stx types under investigation in real serum samples. On the same substrate, droplets of AbStx1 and AbStx2, each at a concentration of 10 μg/mL, were deposited. Subsequently, 0.1 ng/mL of each toxin was applied to its corresponding antibody, as described in Section 2.8.3. Multiple measurements were conducted on each droplet, both before and after the deposition of the Stxs. Figure 6a compares the average SERS spectra for AbStx1 (black line) and Stx1 on its antibody (pink line). Similarly, Figure 6b compares the average spectra for AbStx2 (black line), Stx2a (green line), and cleaved Stx2a (light blue line) on AbStx2.

**Figure 6:**
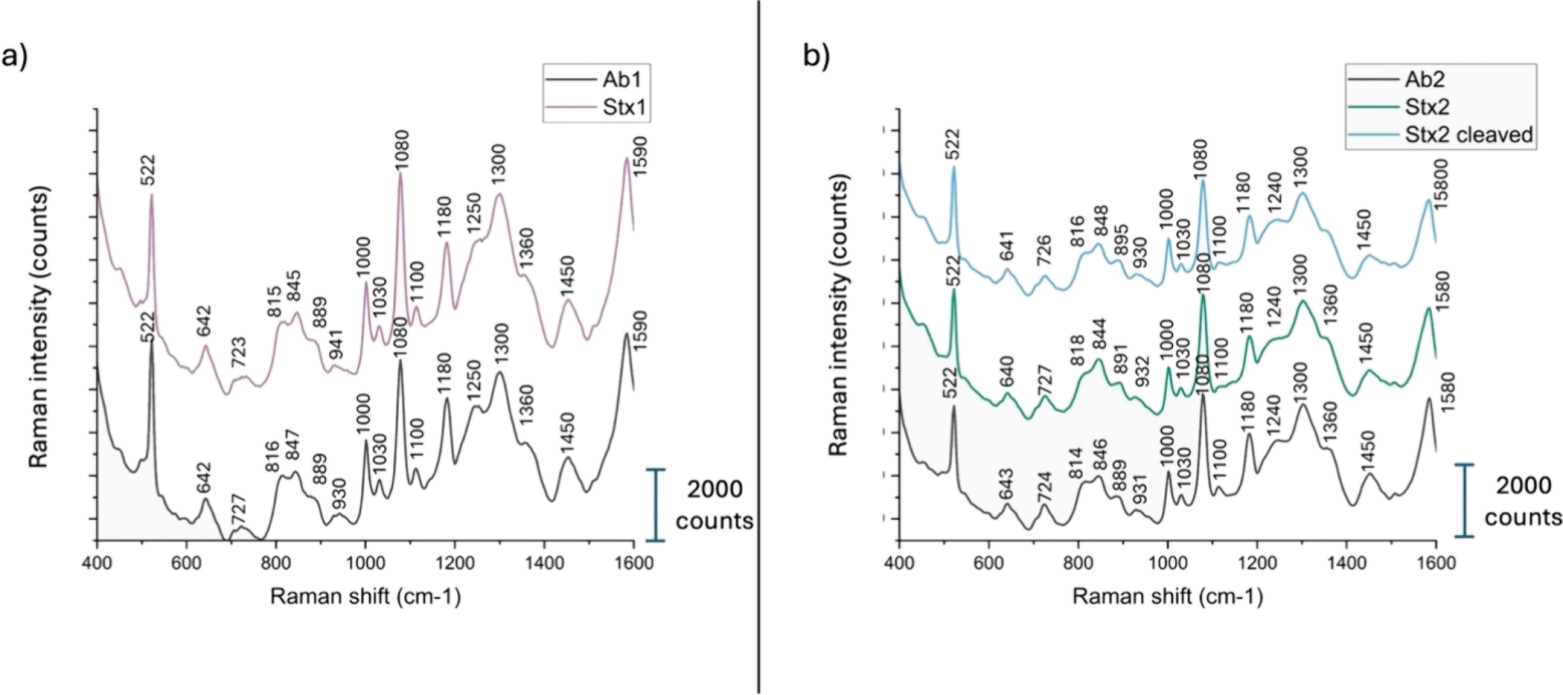
SERS spectra show the interaction of antibodies with toxins in serum. (a) Spectrum of antibody Ab1 physically adsorbed on the SERS substrate (black line), and in the presence of Stx1 in serum (pink line). (b) Spectra of antibody Ab2 physically adsorbed on the SERS substrate (black line), and in the presence of Stx2a (green line) and cleaved Stx2a (light blue line) in serum.

We also applied PCA to discriminate between the three toxin types (see Figure 7). While the antibodies used are specific and inherently serve as discriminating factors, Stx2a and cleaved Stx2a share the same antibody, adding complexity to the analysis. In this scenario, PCA becomes indispensable, as it allows us to effectively differentiate the spectra even when the same antibody is involved, providing an additional layer of accuracy in toxin identification.

**Figure 7:**
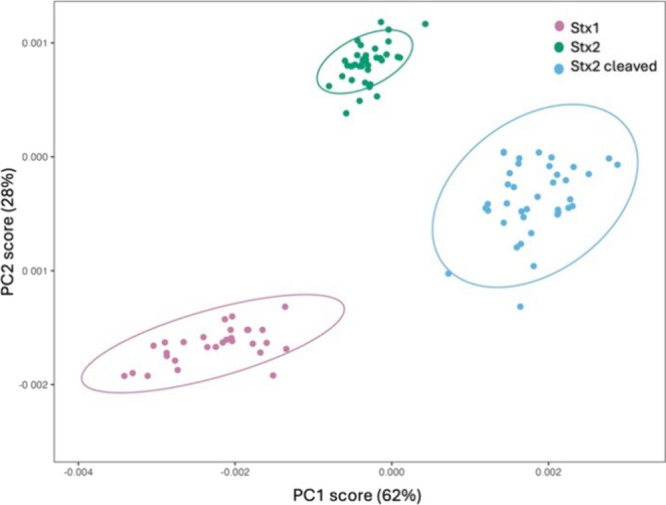
PCA of the SERS spectra for the three toxins bound to their respective antibodies.

Figure 7 presents the PCA score plot for the SERS spectra of Stx1, Stx2a, and cleaved Stx2a, each detected using antibodies immobilized via physical adsorption. The first principal component (PC1) captures 62% of the variance, while the second (PC2) accounts for 28%. The plot reveals a clear separation among the three toxin groups, even at very low concentrations and in a complex matrix. Stx1 (pink) forms a distinct cluster, well-separated along PC1 from both Stx2a (green) and cleaved Stx2a (light blue). Furthermore, Stx2a and cleaved Stx2a are also distinctly separated, with no overlap between their clusters. These results demonstrate that PCA is an effective method for discriminating between the spectral profiles of the toxins, even under conditions where antibodies are immobilized through physical adsorption.

The validation of the PCA model, designed to discriminate among the three Stxs (Stx1, Stx2a, and cleaved Stx2a), yielded highly promising results based on k-means clustering and ANOVA analysis. The k-means clustering analysis (Figure 8) demonstrated strong discrimination between the toxin types, with minimal misclassification (Table 3). Specifically, Stx1 and Stx2a were accurately distinguished from cleaved Stx2a, showing high clustering accuracy and a clear separation between the groups. Only minor overlap occurred, indicating that the PCA model effectively captures distinct spectral features relevant to each toxin.

**Figure 8:**
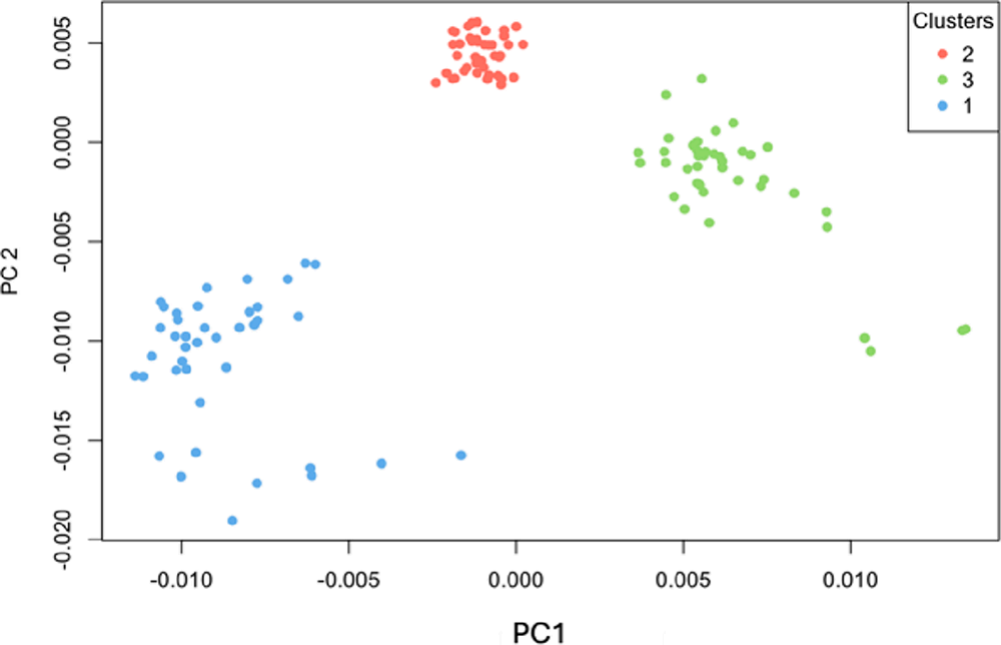
K-cluster analysis applied to the PCA of the SERS spectra for the three toxins bound to their respective antibodies.

**Table 3:** Cluster Assignment of Toxin Spectra on Antibodies

	Stx1	Stx2	Cleaved Stx2
1	0	38	0
2	0	0	38
3	32	2	2

The ANOVA results for both PC1 and PC2 further validate the effectiveness of the PCA model. For PC1, the analysis revealed a highly significant effect of the group variable (F^2, 109^ = 461.6, *p* < 2e^–16^), indicating that PC1 captures substantial variance between the toxin types. Similarly, PC2’s group effect was also significant (F^2, 109^ = 335.6, *p* < 2e^–16^), suggesting that PC2 adds further discriminatory power.

We determined the LOD to evaluate the developed sensing system’s performance in detecting Stx2a. As reported in Figure 9, we measured SERS spectra of the toxin in serum at varying toxin concentrations using sensors functionalized with 10 μg/mL of the Stx2 antibody. For each concentration, 40 individual measurements were taken, and the Raman intensity was averaged to ensure a robust and reliable value. Additionally, to validate the repeatability of the experiment, three independent experimental replicates were performed on different days using sensors from different batches, showing consistent results across trials.

**Figure 9:**
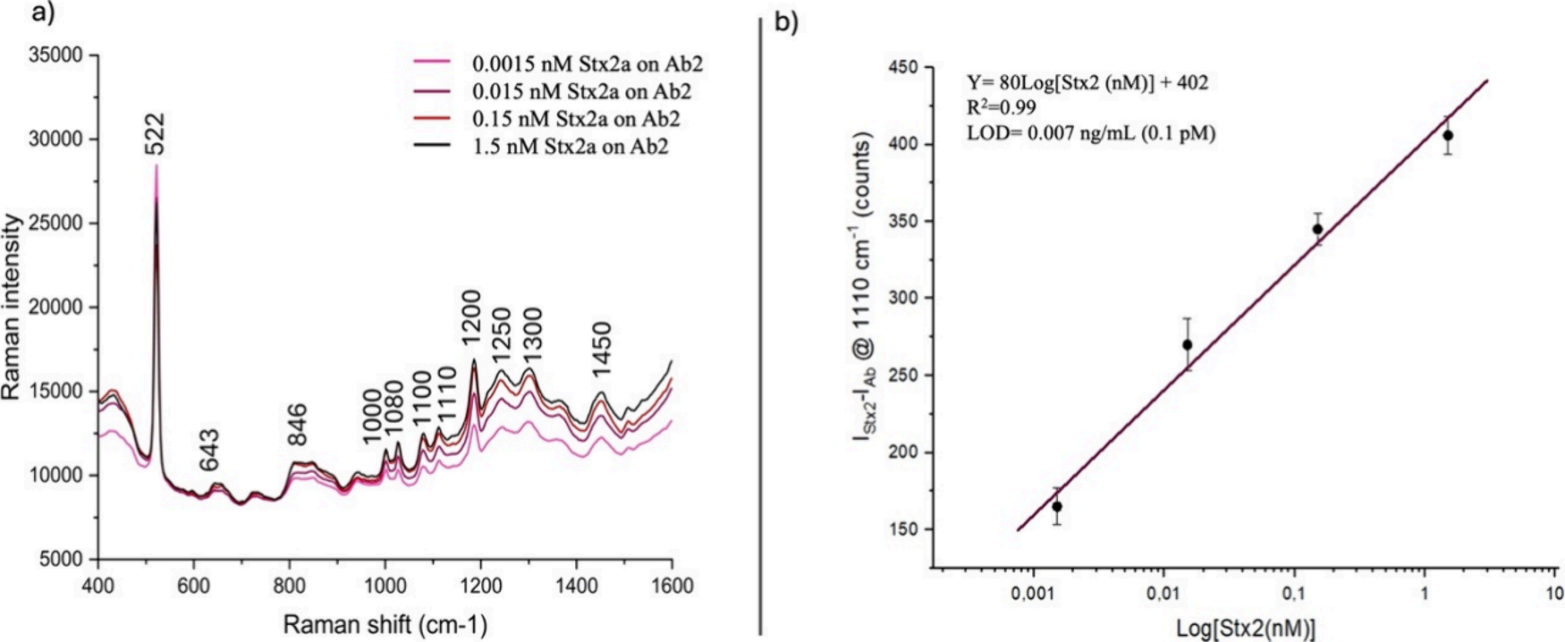
SERS measurements of the Stx LOD performed with the immunosensor. (a) Comparison between the spectra of the Ab + Stx2a complex at increasing concentrations of Stx2a in serum in the range 0.0015–1.5 nM. (b) The calibration curve was achieved by plotting the 1110 cm^–1^ peak intensity (black dots) vs the toxin concentrations and linear fit associated with data (violet line).

The sensor’s performance in detecting Stx2a was evaluated by analyzing the Raman intensity at 1110 cm^–1^. As shown in Figure 9a, the SERS signal intensity increases with higher toxin concentrations, demonstrating a clear dose-dependent response. This trend confirms the sensitivity of the antibody-functionalized sensor in detecting varying concentrations of the toxin. The relationship observed between the SERS intensity and the toxin concentration further validates the quantitative capabilities of the developed system.

In Figure 9b, the *y*-axis shows the net SERS intensity of the toxin’s peak at 1110 cm^–1^, obtained by subtracting the intensity of this peak for the antibody alone from that measured for the toxin bound to the antibody. The experimental data points exhibit a good linear relationship between the intensity and the logarithmic values of the toxin concentration. The best-fit equation, i_Stx2a_-I_Ab2_=a Log­[I_Stx2a_]+b, was obtained with a = 80, b = 402, and an r^2^ value of 0.99 (violet line in Figure 9b). The LOD was calculated by intersecting the equation with the 3σ threshold, where σ represents the standard deviation of the blank control, which was calculated to be 27.^58^ Using this approach, the LOD for Stx2a was determined to be 0.007 ng/mL. To enhance the robustness of our findings, the experimental reproducibility was further assessed by calculating the Relative Standard Deviation (RSD%) for each concentration. This metric was used to quantify the variability of the measurements and assess the precision of the sensor system. The obtained RSD% values were 9.9% for 0.1 ng/mL, 8.0% for 1 ng/mL, 10.3% for 10 ng/mL, and 10.5% for 100 ng/mL, confirming a good level of repeatability even at low concentrations. The low RSD% values across replicates and concentrations confirm both intra- and intersample reproducibility, supporting the robustness of the developed sensor. To the best of our knowledge, our estimated LOD value for the system proposed here is much lower than those reported in the literature.^27^ In Table 4, we summarize the key features and LODs of representative diagnostic methods for Shiga toxin detection. Our method outperforms both conventional techniques (such as PCR, Lateral Flow Immunoassay, and ELISA) and emerging approaches (including LSPR and other SERS-based devices), highlighting its superior sensitivity. Additionally, our platform offers several other advantages, including label-free detection, the ability to distinguish between structurally similar toxin subtypes (Stx1, Stx2, and cleaved Stx2), and excellent signal reproducibility.

**Table 4:** Current Technologies for Detecting Shiga Toxins from *E. coli*

Technique	Feature	Analyte	LOD	ref.
LSPR platform	Synthetic glycosyl ceramides attached to gold nanoparticles	Stx	10 ng/mL	^59^
LSPR platform	Gold nanoparticles bound to antibodies	Stx2	10 ng/mL	^60^
SPC-ECL	Nanospheres of Au NPs encapsulated into a solid silica core with graphite phase carbon nitride quantum dots embedded	Stx	9 fM	^61^
SPRi	Biochip with 50 nm gold film	Stx1, Stx2	50 ng/mL	^31^
SERS substrate	Recycled silicon chips	Stx2	0.3158 μM	^12^
SERS device	Noble metal nanoparticles and Raman reporter loaded metal–organic framework	Stx2	0.82 ng/mL	^22^
LC-MS	Nanospray liquid chromatography–mass spectrometry and Vero cell bioassay	Stx	Stx2c (5.7 ng/mL)	^62^
Reverse Latex Agglutination	VTEC-Screen Seiken	Stx1, Stx2	25 ng/mL	^63^
Nanoparticle Platform	Gold nanoparticles conjugated with Gb3 and silver enhancement	Stx1	1 μg/mL	^64^
Nanoparticle Platform	Magnetic nanoparticles conjugated with Gb3 and MALDI-TOF	Stx1	330 pg/mL	^65^
(mPCR) assay	Vero cells in a three-dimensional (3D) platform	Stx1, Stx2	32 ng/mL	^66^
Lateral Flow Immunoassay	Based on AuNP and CdTe QD	Stx2	25 ng/mL	^67^
SERS device	2D hybrid metallic polymeric nanostructure based on the octupolar framework	Stx2	1.4 nM	^29^
AlphaLISA	Bead-based immunoassay	Stx	0.5 ng/mL	^62^

Our study effectively demonstrates the detection of toxins in controlled laboratory settings. Potential clinical deployment challenges such as differences in serum composition among patients or interference from other biomolecules seem unlikely since the main Stx2-binding molecule found in human sera (serum amyloid P component) is present at a relatively stable concentration in blood and does not bind Stx1.^68^ Clinical validation strategies aiming at comparing sera from many different donors after Stx addition might be easily performed to reveal the effect of minor Stx-binding molecules, if present. The final challenge would be the application of our method to the detection of the low amounts of Stx bound to extracellular vesicles derived from blood cells during the crucial step of HUS pathogenesis. This seems feasible given the low limit of detection calculated in the present paper. Indeed, compared to other reported approaches for Shiga toxin detection (Table 4), our method demonstrates a significantly improved LOD (0.007 ng/mL), which is among the lowest reported to date for SERS-based sensors. Additionally, unlike conventional immunoassays or most SERS systems, our platform enables discrimination between different toxin subtypes (Stx1, Stx2, and cleaved Stx2) directly in human serum. This highlights both the sensitivity and the diagnostic relevance of the system, especially in complex biological environments. Finally, a diagnostic system based on the proposed would be suitable to produce miniaturized chips and portable readers equipped with interpretation systems based on automated analyses to be used at the point of care. Such a diagnostic would dramatically improve the diagnosis of STEC infections allowing a better patient’s management.

To assess the specificity of the developed SERS-based assay for Stx detection, a series of control experiments were performed using both a nontarget protein and complex biological matrices. First, the Stx2a antibody was incubated with the lectin Erythrohemagglutinin PHA-E (*Phaseolus vulgaris*), used as a negative control at two concentrations (1.047 × 10^4^ ng/mL and 0.1 ng/mL). As shown in Figure 10a, no increase in signal intensity was observed at either concentration, and the spectra were fully consistent with those of the antibody alone, indicating the absence of cross-reactivity with unrelated glycoproteins. To further evaluate potential matrix effects and nonspecific interactions, additional tests were carried out using human serum. Each antibody was exposed to serum alone and the noncognate toxin (Stx1 on Ab2 and Stx2 on Ab1), both at 154 nM in serum. As shown in Figure 10b–d, the spectra recorded for serum alone and for the noncognate toxin–antibody combinations displayed a minor additional peak (700 cm^–1^) not present in the antibody-only spectra. However, no signal enhancement was detected, in contrast to the clear intensity increase observed for the cognate toxin–antibody interactions (Figure 6). This suggests that the additional peak likely arises from weak, nonspecific adsorption of serum components or minor matrix effects, rather than from a specific binding event. The blocking step with 1% w/w BSA effectively minimized these interactions, as confirmed by the lack of signal amplification in the negative controls. Overall, these results demonstrate the high specificity of the sensing platform, confirming that neither unrelated proteins nor serum components significantly interfere with the antibody–toxin recognition process.

**Figure 10:**
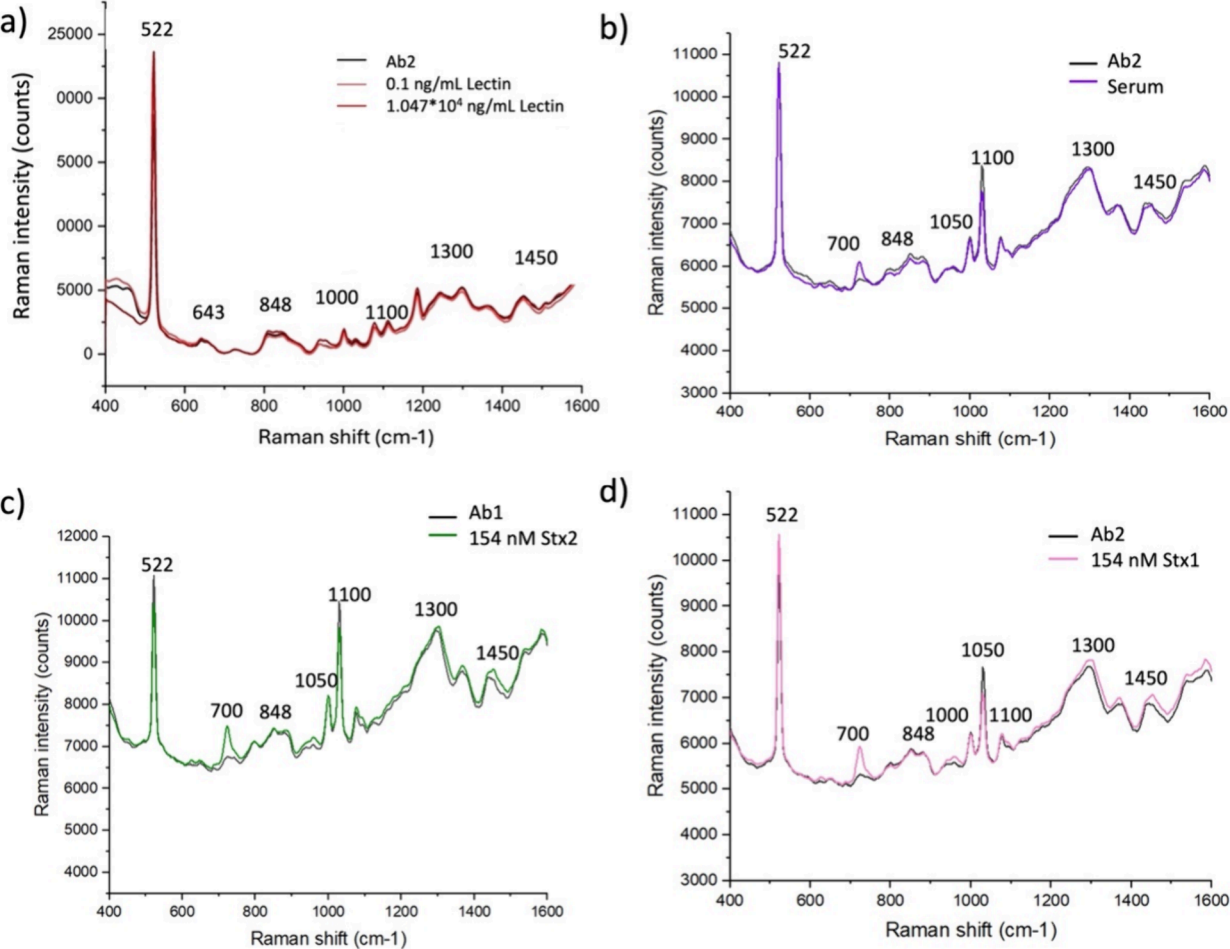
Specificity test. SERS spectra demonstrate the specificity of the antibodies for Stxs. a) Spectra of the antibody alone and in the presence of lectin at two concentrations (1.047 × 10^4^ ng/mL and 0.1 ng/mL). b) Spectra of the antibody alone and in the presence of serum. c-d) Spectra of antibodies alone and in the presence of noncognate toxins in serum (Stx2 on Ab1 in panel c and Stx1 on Ab2 in panel d, both at a concentration of 154 nM).

### 3.5 Proof of Concept in Human Serum: Detection of Stx in HUS Patients

In the present paper, we aimed to provide a preliminary proof-of-concept validation of the platform using a limited number of clinical samples. To this end, we analyzed sera from four patients with HUS previously confirmed by RT-PCR for the presence of *stx1* and/or *stx2* genes in feces. The clinical features of the HUS patients and the laboratory tests used for the diagnosis of STEC infection are shown in Table 5.

**Table 5:** Clinical Features of the HUS Patients Enrolled in the Study

Patients	Age (y)	Gender	Diarrhea	Bloody diarrhea	HUS	Neurological complications	Dialysis	Red cells transfusions	*E. coli* serotype^a^	Detection of stx genes in feces (RT-PCR)^b^	Serum sampling time^c^	Detection of Stx in serum (Biosensor)
1	2.3	F	+	+	+	-	+	+	O26	*stx1* + *stx2*	early	Stx1 + Stx2
2	2.3	M	+	+	+	-	+	+	O157	*stx2*	early	Stx2
3	3	F	+	+	+	+	+	+	O103	*stx2*	late	undetectable
4	0.9	M	+	+	+	-	-	+	O26	*stx2*	late	undetectable

a *E. coli* serotypes were determined by stool cultures and serotype analysis.

b Multiplex Real-Time PCR (R-Biopharm) for the main virulence genes (*eae*, *stx1*, *stx2*) was performed.

c Sera were collected 2 days (early sampling) or 5–7 days (late sampling) after the detection of Stx in feces (RT-PCR).

The first problem to tackle was the individual variability of patients’ sera since the PCA-based discrimination described above is effective only when spectra are acquired from the same serum background. For this reason, each clinical sample was tested in parallel with spiked positive controls carried out by adding toxins to patients’ sera. The assay was performed under blinded conditions as the toxin profile of the clinical samples was unknown during spectral acquisition and analysis. Two serum samples were collected 2 days after the diagnosis of STEC infection (Table 5) and derived from patients infected by strains producing both Stx1 and Stx2 (patient 1) or only Stx2 (patient 2). PCA analysis of spectra obtained using the anti-Stx2 antibody revealed a complete overlap between the patient samples and their corresponding spiked controls, indicating the presence of Stx2 in the unmodified serum of both patients, despite the expected low levels. This finding aligns with clinical data indicating that both patients had developed HUS, a condition strongly associated with Stx2 exposure (Figure 11).

**Figure 11:**
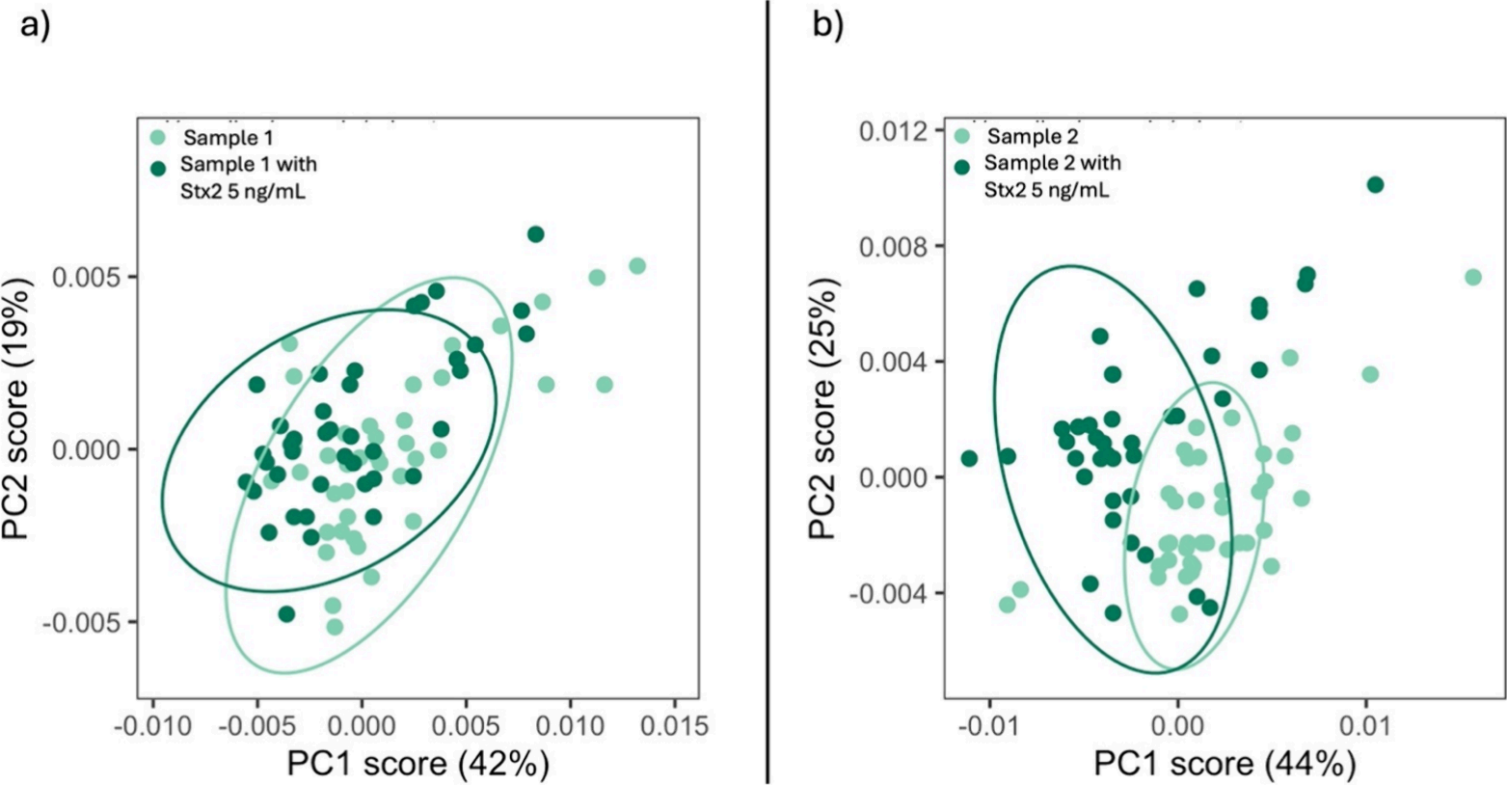
PCA with Ab2 (anti-Stx2). a) Patient sample 1 vs the same sample spiked with Stx2 (5 ng/mL): the two groups completely overlap, suggesting that the real sample already contains Stx2. b) Patient sample 2 vs the same sample spiked with Stx2 (5 ng/mL): again, the two groups completely overlap.

In contrast, when using the anti-Stx1 antibody (Figure 12), sample from patient 1, known to contain Stx1, produced spectra that fully overlapped with its corresponding positive control, confirming the presence of Stx1. In the second sample (patient 2), partial overlap was observed between the unspiked serum and its Stx1-spiked counterpart, likely due to the shared serum matrix, the presence of Stx2, and the relatively low concentration of toxin used for spiking. This result is consistent with the expectation that Stx1 is absent in that sample.

**Figure 12:**
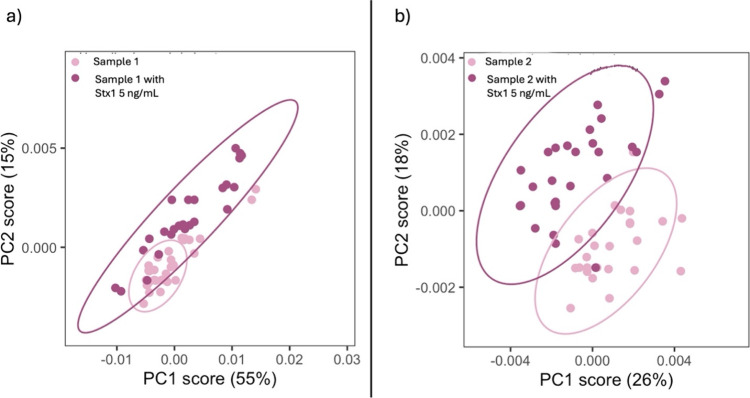
PCA with Ab1 (anti-Stx1). a) Patient sample 1 vs the same sample spiked with Stx1 (5 ng/mL): complete overlap, suggesting the presence of Stx1 in the real sample. b) Patient sample 2 vs the same sample spiked with Stx1 (5 ng/mL): only partial overlap, indicating the absence of Stx1 in the real sample.

Conversely, two serum samples (patients 3 and 4) were obtained 5–7 days after the detection of *stx2* genes in feces during the late phase of STEC infection when the amount of toxins in blood is negligible.^69^ As expected, in those cases, the PCA revealed separation from the Stx2-spiked control, suggesting the absence of the toxin. This observation further supports the consistency of the SERS-based assay. Although the spectral data from the positive clinical samples (patients 1 and 2) were not projected onto the PCA space reported in section—due to differences in serum origin and composition—the observed clustering behavior is encouraging. It suggests that our platform can detect both Stx1 and Stx2 in real patient-derived matrices. These findings support the potential of our method as a basis for more advanced machine learning models capable of generalizing across interpatient variability in serum background, thus enabling robust and clinically meaningful detection of Shiga toxins.

## 4 Conclusions

This study demonstrates a fast, cost-efficient bottom-up method to fabricate high-quality SERS substrates via self-assembly of citrate-stabilized gold nanoparticles at liquid–liquid interfaces. These substrates exhibit exceptional sensitivity, enabling detection of Shiga toxins at picomolar levels directly in human serum, while clearly distinguishing structural variants (Stx1, Stx2, cleaved Stx2). With a limit of detection of 0.007 ng/mL—among the lowest reported for SERS sensors—our platform combines high sensitivity with diagnostic relevance. By integrating SERS spectroscopy with multivariate statistical methods such as PCA and k-cluster analysis, we established a robust framework for toxin classification, addressing the challenges posed by similar spectroscopic signatures in complex environments. This approach enhances diagnostic sensitivity and specificity, offering a scalable and versatile platform for clinical and environmental applications. To highlight the relevance of rapid and sensitive detection of Stx in patients, it should be noted that, in children infected by STEC, the risk of developing HUS depends on the Stx type and not on the STEC serogroup; consequently, it is difficult to quantify the risk with indeterminate Stx results.^1^ Future improvements in substrate design and the standardization of functionalization protocols could further enhance reproducibility and scalability. Overall, this work lays the foundation for the rapid and reliable detection of critical biomarkers, advancing the diagnosis and management of infections caused by toxigenic *Escherichia coli.*

To further validate the real-world applicability of our platform, we analyzed serum samples from patients diagnosed with HUS in the late stage of the disease. Despite the anticipated low concentration of circulating toxins at this stage, our SERS-based system, combined with subtype-specific antibodies and multivariate analysis, was able to detect and differentiate Stx1 and Stx2 directly in clinical matrices. Notably, the discrimination between native and spiked samples was successful even at low toxin concentrations, underscoring the platform’s high sensitivity and robustness. These promising results represent a critical step toward clinical translation and highlight the potential of our sensor to operate effectively in highly variable, patient-derived samples. Future work will focus on training supervised machine learning models on larger, multidonor data sets to extend the exploratory analyses and account for interpatient variability. While PCA proved highly effective for toxin discrimination in complex matrices, it does not allow consistent classification across different serum samples. Therefore, more advanced supervised algorithms will be required to achieve reliable generalization and quantitative evaluation. Incorporating proper cross-validation and external test sets will ensure model robustness and enable reliable toxin classification in new serum samples. Ultimately, integration with portable, AI-assisted readers could enable on-site diagnosis during the early stages of infection, supporting timely therapeutic decisions and reducing the risk of HUS progression.

## Supplementary Material



## Data Availability

The data sets generated and/or analyzed during the current study are available from the corresponding author upon reasonable request.

## Notes

Substrates’ enhancement factor, plasmonic characterization by LSPR reflectance spectroscopy, cost analysis and comparison of the SERS substrate (PDF)

## Abbreviations

Stx: Shiga toxin
STEC: Shiga-toxin producing *E. coli*
HUS: Hemolytic uremic syndrome
SERS: Surface-enhanced Raman spectroscopy
PCA: Principal component analysis
4MBA: 4-Mercaptobenzoic acid
LOD: Limit of detection
PBS: Phosphate-buffered saline
BSA: bovine serum albumin
EF: Enhancement factor
LSPR: Localized surface plasmon resonance
SI: Supporting information
LB: Langmuir–Blodgett
RDS%: Relative standard deviation
