# Supplementary material for: Advancing Medical Diagnostics: Rapid, Label-Free Detection and Differentiation of Shiga Toxin Variants in Human Serum Using a Cost-Effective PCA-Assisted SERS Platform
Source: ACS Appl Mater Interfaces. 2025 Nov 7;17(46):63237–52. doi: 10.1021/acsami.5c18171 (PMC12635968; doi:10.1021/acsami.5c18171)
Supplement: Supplementary file 1 [file am5c18171_si_001.pdf]

**Supporting Information**

# Advancing Medical Diagnostics: Rapid, Label-Free Detection and Differentiation of Shiga Toxin Variants in Human Serum Using a Cost-Effective PCA-Assisted SERS Platform

Alessia Milano[1‡], Amalia D'Avino[1,2‡], Valentina Marchesano[1*], Domenico Sagnelli[1], Massimo Rippa[1], Bryan Guilcapi[1], Lu Zhou[3], Elisa Varrone[4], Giorgia Rossi[4], Maurizio Brigotti[4*], Gianluigi Ardissino[5], Stefano Morabito[6], and Lucia Petti[1*]

[1] Institute of Applied Sciences and Intelligent Systems "E. Caianiello" CNR, Pozzuoli, 80078, Italy
[2]Department of Engineering, University of Naples "Parthenope", Centro Direzionale Isola C4, Napoli, 80143, Italy
[3] Shandong First Medical University & Shandong Academy of Medical Sciences, Jinan, 271016, China
[4]Dipartimento di Scienze Mediche e Chirurgiche, Sede di Patologia Generale, Università di Bologna, Bologna, 40126, Italy
[5] Fondazione IRCCS Ca' Granda Ospedale Maggiore Policlinico, Milano, 20122, Italy
[6]Department of Food Safety, Nutrition and Veterinary Public Health, Istituto Superiore di Sanità, Rome, 00161, Italy

*Corresponding authors, E-mail: l.petti@isasi.cnr.it; maurizio.brigotti@unibo.it; valentina.marchesano@cnr.it

‡The authors contributed equally to the work

**Index**

1. Plasmonic characterization by LSPR reflectance spectroscopy
2. Simulation AuNPs.
3. Substrates' enhancement factor
4. Cost analysis and comparison of the SERS substrate

## 1. Plasmonic characterization by LSPR reflectance spectroscopy

To further evaluate the plasmonic properties of the fabricated SERS platform, Localized Surface Plasmon Resonance (LSPR) reflectance spectroscopy was performed using a custom-built optical setup[1] (Figure S1). This technique enables the estimation of the resonance wavelength associated with the collective oscillation of conduction electrons in the gold nanoparticle (AuNP) monolayer, which directly influences SERS efficiency. A broadband white light laser source was directed onto the self-assembled AuNP monolayer deposited on a silicon substrate. The reflected light was collected via a fiber-optic cable and analyzed using a USB4000 spectrometer (Ocean Optics).

Reflectance spectra were recorded in the 400–850 nm range[2], using a bare silicon substrate as the reference. The reflectance percentage spectrum is shown in Figure S2.

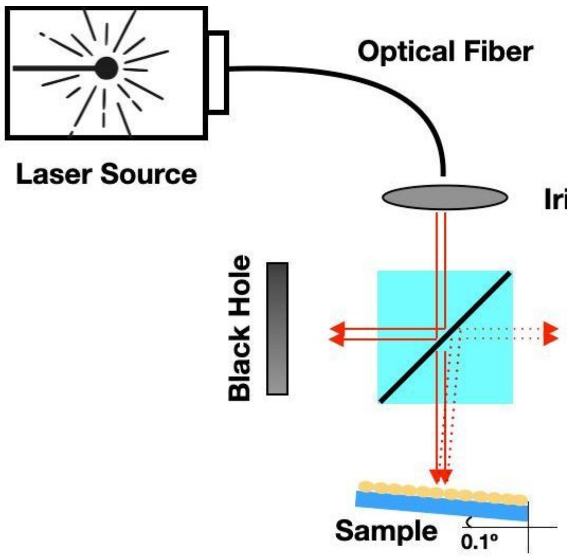


**Figure S1**. Reflectance Spectroscopy set-up.

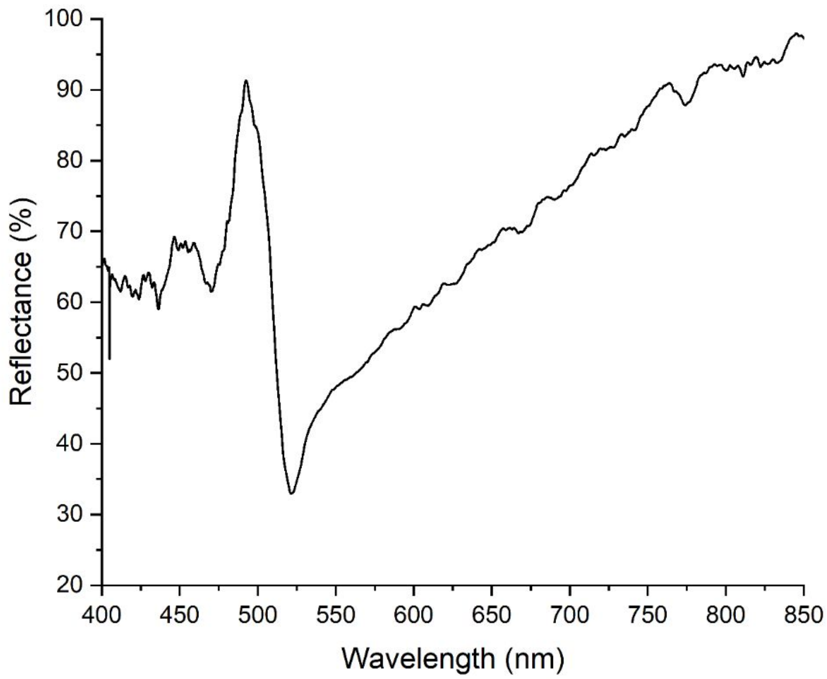


**Figure S2**. Experimental reflectance spectrum R(%) of the AuNP monolayer substrate.

## 2. Simulation AuNPs

We performed Finite-Difference Time-Domain (FDTD) simulations using a Total Field / Scattered Field (TFSF) source to calculate the squared magnitude of the electric field. Raman scattering is an inelastic scattering of a photon, meaning that scattered photons will have different frequencies from

the excitation. When the scattering molecules are on a textured surface, the Raman scattering can be greatly enhanced (thus the term Surface Enhanced Raman scattering (SERS)[3,4].

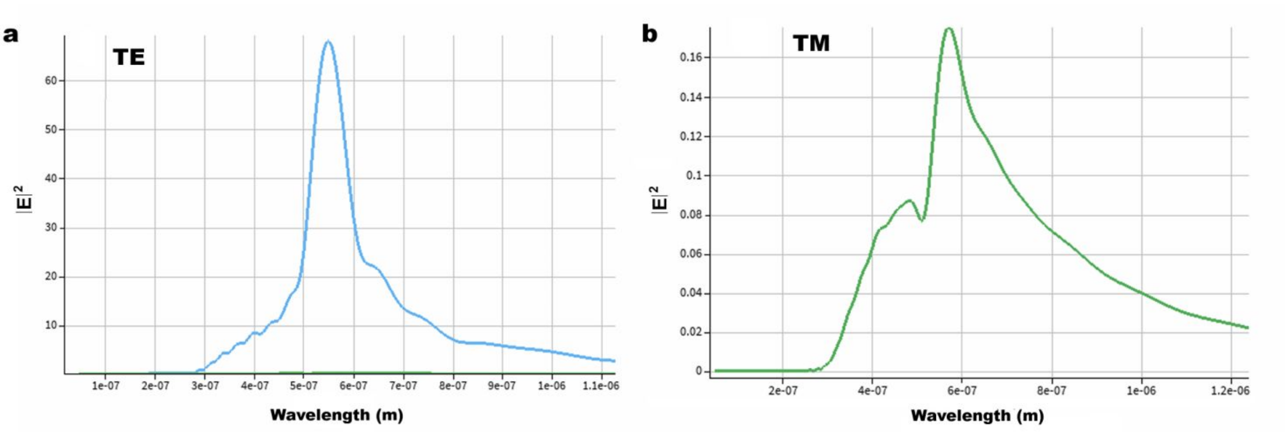


**Figure S3.** FDTD simulations: |E(λ)|2 spectra calculated for (a) TE- and (b) TM-polarized components

A single AuNP (e.g., diameter~43nm) on a Si substrate is simulated with a TFSF source. Monitors record near fields (E,H) and spectral power. Key materials use dispersive models (e.g., Drude–Lorentz for Au; tabulated Si).

Spectral fields (Figure S3). The electric field intensity $|E(\lambda)|^2$ sampled at the particle–substrate junction exhibits a maximum near the LSPR (consistent with the experimental reflectance valley). The magnetic field spectrum is co-modulated but typically less localized than the electric hot spot.

Near-field enhancement. In the dipolar regime, the maximum field at the particle surface occurs near LSPR. A common SERS scaling is

$G_{SERS}(\lambda_{exc}, \lambda_{sc}) \alpha \ |E(\lambda_{exc})|^2 |E(\lambda_{sc})|^2 \approx |E(\lambda_{LSPR})|^4$ (If excitation and Stokes lie near LSPR)

- TE-polarized component: field enhancement peak ~ 540 nm
- TM-polarized component: field enhancement peak ~ 590 nm

## 3. Substrates' enhancement factor

To assess the plasmonic performance of the fabricated SERS substrates, we estimated the SERS Enhancement Factor (EF) using 4MBA as a probe molecule. 4MBA was selected due to its well-defined and strong Raman signature, making it a widely used standard in SERS studies. The EF provides a quantitative measure of the signal amplification achieved by the substrate, which is critical for evaluating its suitability for sensing applications.
To estimate the SERS EF, we compared the SERS spectrum of 4MBA with its conventional Raman spectrum acquired on a silicon wafer. The EF resulting approximately $3.13 \times 10^7$, was calculated using the standard formula: EF= ($I_{SERS}/I_{bulk}$) X ($N_{bulk}/N_{SERS}$), where $I_{SERS}$ and $I_{bulk}$ are the integrated intensities of the 1079 $cm^{-1}$ peak in the SERS and bulk Raman spectra, respectively. In the SERS and bulk measurements, $N_{SERS}$ and $N_{bulk}$ represent the number of probed molecules under the laser spot. The laser spot diameter was estimated as 1.28 µm using the equation: D=( λ/NA) X 1.22, where λ = 785 nm and NA= 0.75, with the 1.22 factor accounting for beam deviation from an ideal Gaussian

profile. Following our previously established protocol, $N_{SERS}$ was calculated as 3.9× $10^6$ using: $N_{SERS}$= ($N_A$*A)/ σ, where $N_A$ is Avogadro's number, A is the effective laser-irradiated area, and σ=2.0×109 $cm^2$ $mol^{-1}$ is the area occupied by a self-assembled monolayer of 4MBA. For the bulk measurement, $N_{bulk}$ was estimated to be 2.7 X $10^{11}$, based on a density of 1.5 g·$cm^{-3}$ and a molecular weight of 154.19 g·$mol^{-1}$ for 4MBA. From the experimental spectra, $I_{SERS}$ and $I_{bulk}$ were measured as 1.9 X $10^6$ and 4.2 X $10^3$, respectively. This yields an EF of approximately 3.13 × $10^7$, indicating a strong enhancement performance of the fabricated SERS substrate (Figure S4). To assess signal uniformity, SERS spectra were collected from 40 randomly selected points across the substrate. The spectra were highly consistent, showing negligible variation in intensity or peak position, confirming the spatial uniformity and reproducibility of the plasmonic enhancement.

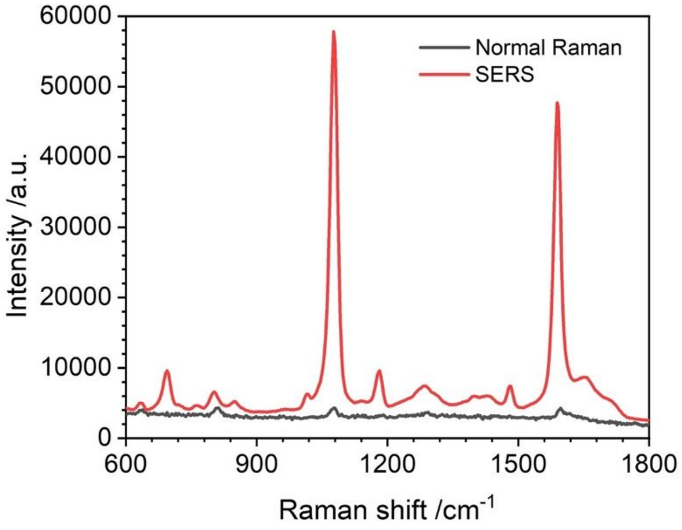


**Figure S4.** Raman (black) and SERS (red) spectra of 4MBA powder on the substrate and 4MBA solution (10mM) on the gold nanoparticles layer attached to silicon, respectively.

## 4. Cost analysis and comparison of the SERS substrate

The total material cost for fabricating a single SERS substrate using our Langmuir–Blodgett self-assembly method was estimated to be € 4.23. This includes the cost of gold nanoparticles, silicon wafers, and all consumables. Considering personnel time and laboratory processing, the overall cost per substrate is approximately €15. Notably, the fabrication procedure allows the preparation of up to 6 substrates per monolayer deposition, improving cost-efficiency for batch production.
Compared with commercially available SERS substrates, our sensor demonstrates a significant cost advantage. The market prices for single-use substrates range from €30 to €60, as reported by manufacturers such as Silmeco (€60), ThorLabs (€43.77), FindLight (€30), SERSitive (€50), and ATOID (€58.63). Although some literature examples report lower fabrication costs [5–7], these are typically based on paper substrates or low-cost materials that may lack the reproducibility, structural control, and robustness required for quantitative analyses in complex matrices [8–12]. In contrast, our substrate combines the low material cost of €4.23 with the structural uniformity and reproducibility enabled by the Langmuir–Blodgett self-assembly of gold nanoparticles onto silicon wafers. Moreover, the ability to prepare multiple substrates in a single monolayer deposition enhances scalability and reduces cost per unit in batch production. Including processing and personnel time, the overall cost of €15 per substrate remains competitive, especially considering the performance, stability, and reproducibility advantages of our silicon-based nanostructured platform.

References


(1) Mcoyi, M. P.; Mpofu, K. T.; Sekhwama, M.; Mthunzi-Kufa, P. Developments in Localized Surface Plasmon Resonance. *Plasmonics*. Springer July 1, 2025, pp 5481–5520. https://doi.org/10.1007/s11468-024-02620-x.

(2) Schiattarella, C.; Sanità, G.; Guilcapi Alulema, B.; Lanzio, V.; Cabrini, S.; Lamberti, A.; Rendina, I.; Mocella, V.; Zito, G.; Romano, S. High-Q Photonic Aptasensor Based on Avoided Crossing Bound States in the Continuum and Trace Detection of Ochratoxin A. *Biosens Bioelectron X* 2022, *12*. https://doi.org/10.1016/j.biosx.2022.100262.

(3) VanDeHulst_Light_scattering_by_small_particles_1981R.

(4) Bohren, C. F. .; Huffman, D. R. . *Absorption and Scattering of Light by Small Particles*; Wiley-VCH, 2009.

(5) Li, H.; Dumont, E.; Slipets, R.; Thersleff, T.; Boisen, A.; Sotiriou, G. A. Democratizing Robust SERS Nano-Sensors for Food Safety Diagnostics. *Chemical Engineering Journal* 2023, *470*. https://doi.org/10.1016/j.cej.2023.144023.

(6) Verma, M.; Naqvi, T. K.; Tripathi, S. K.; Kulkarni, M. M.; Dwivedi, P. K. Paper Based Low-Cost Flexible SERS Sensor for Food Adulterant Detection. *Environ Technol Innov* 2021, *24*. https://doi.org/10.1016/j.eti.2021.102033.

(7) Restaino, S. M.; White, I. M. A Critical Review of Flexible and Porous SERS Sensors for Analytical Chemistry at the Point-of-Sample. *Analytica Chimica Acta*. Elsevier B.V. July 4, 2019, pp 17–29. https://doi.org/10.1016/j.aca.2018.11.057.

(8) Lin, S.; Lin, X.; Han, S.; Liu, Y.; Hasi, W.; Wang, L. Flexible Fabrication of a Paper-Fluidic SERS Sensor Coated with a Monolayer of Core–Shell Nanospheres for Reliable Quantitative SERS Measurements. *Anal Chim Acta* 2020, *1108*, 167–176. https://doi.org/10.1016/j.aca.2020.02.034.

(9) Naqvi, T. K.; Bajpai, A.; Dwivedi, S.; Bhaiyya, M.; Goel, S.; Dwivedi, P. K. Flexible, Label Free and Low-Cost Paper Based Microfluidic SERS Substrates for Thiram Detection. *Sens Actuators A Phys* 2023, *356*. https://doi.org/10.1016/j.sna.2023.114341.

(10) Mahanty, S.; Majumder, S.; Paul, R.; Boroujerdi, R.; Valsami-Jones, E.; Laforsch, C. A Review on Nanomaterial-Based SERS Substrates for Sustainable Agriculture. *Science of the Total Environment*. Elsevier B.V. November 10, 2024. https://doi.org/10.1016/j.scitotenv.2024.174252.

(11) Naqvi, T. K.; Gupta, A.; Parab, D.; Dwivedi, P. K. Low Cost Disposable Paper SERS Substrate for Explosive Detection. *Sens Actuators A Phys* 2025, *389*. https://doi.org/10.1016/j.sna.2025.116533.

(12) Liu, C.; Xu, D.; Dong, X.; Huang, Q. A Review: Research Progress of SERS-Based Sensors for Agricultural Applications. *Trends in Food Science and Technology*. Elsevier Ltd October 1, 2022, pp 90–101. https://doi.org/10.1016/j.tifs.2022.07.012.
